# Born to act: Deferred action and desire as active inference

**DOI:** 10.3758/s13415-025-01334-9

**Published:** 2025-08-04

**Authors:** Valery Krupnik

**Affiliations:** Department of Mental Health, US Naval Hospital Camp Pendleton, H200 Mercy Circle, Camp Pendleton, CA 92055 USA

**Keywords:** Motivation, Desire, Deferred action, Active inference, Self-efficacy, Obsessive-compulsive disorder, Depression

## Abstract

The active inference framework (AIF) considers the brain as a generative model guiding behavior under the imperative of minimizing the model’s variational free energy. Computationally, this is accomplished by hierarchical Bayesian inference. The theory views organisms as doxastic agents, which has drawn the criticism of being insufficient to explain conative agents motivated by desire. Specifically, it has been noted that the concept of desire is not isomorphic with belief and, therefore, fits poorly with AIF. In this paper, we build on previous work that suggests a path to integrating conation in AIF and present three arguments. First, the dichotomy between belief and desire is unnecessary. To that end, we define desire as a hierarchical inference that starts from a domain-general inference on the agent’s affective dynamics (affective charge) and descends to contextualized inference on the precision of action policies. We suggest that this hierarchy is implemented by a coordinated activity of the intrinsic brain networks: default mode, action mode, executive, and salient. Second, we argue for a central role that deferred action plays in the process of desire by allowing for affect-dependent awareness of the agent’s states over different timescales. Third, we suggest that the proposed model of desire and deferred action has ramifications for understanding psychopathology, which we frame as the malfunction of deferred action and desire and use obsessive-compulsive disorder and depression as examples. This view entails that disorders of affect and motivation are subjectively desired despite their associated suffering.

“If in the first act you have hung a pistol on the wall, then in the following one it should be fired.” A. Chekhov

Motivation is a central phenomenon that psychological sciences aim to explain in order to answer the perennial question of why people do what they do and not otherwise. The predictive processing theories, and specifically active inference (AIF), explain motivation by the need to minimize prediction errors (PE) via minimizing the agent’s variational free energy (Parr et al., 2022).^1^ Such minimization, as stipulated by the free-energy principle (FEP), is imperative for the very existence of self-organizing nonequilibrium systems, which all living organisms are (Friston, 2009). At the same time, the limitations of the prediction error-driven account of motivation have been noted. One is the assumed dichotomy, envisioned in Hume’s philosophy, between belief states, that PE dynamics are concerned with, and desire states (Colombo, 2016). It is argued that AIF is incompatible with Hume’s theory of motivation due to subsuming belief and desire under the unified process of minimizing free energy. The conflation of belief and desire states has been criticized as lacking explanation for certain neurocognitive phenomena, such as compulsions in obsessive-compulsive disorder (Yon et al., 2020). A more fundamental challenge levelled at the predictive processing approach to motivation is that predicting a certain state—even a highly likely one—may not explain why the agent would want to act on this prediction as opposed to merely update the agent’s predictions according to the prediction error (Klein, 2018).

Four interrelated arguments have been advanced in response to these challenges. One is that certain states are prioritized and predicted as highly likely and, therefore, actions that are expected to bring them about are predicted with a high probability, making these actions “desirable.” Such predictions have been called “first priors;” they are innate and correspond to the agent’s phenotype-typical homeostasis-dependent states (Allen & Tsakiris, 2019). First priors can be seen as organizing constraints on the agent’s belief space. The second is that AIF incorporates desire in its formalism (Smith et al., 2022), where the probability of an action is proportional to the agent’s confidence (precision of the belief about the action) that the action will result in the agent’s preferred state. Such states can be considered as desirable, thereby making predictions/beliefs isomorphic with desire. Furthermore, in their simulated agent, the authors illustrate how the strength of desire can be represented by the precision parameter. That the precision parameter can be seen as modelling the strength of a desire is the third argument (also made in Schwartenbeck et al., 2019) for conation’s fit in AIF. The fourth argument advances an enactive-ecological view of conation (Clark, 2020; Kiverstein et al., 2024), where it is considered as a predicted action in the context of environmental affordances as opposed to the more abstract folk-psychological notion of drive. The strength of desire is thought to be controlled by the balance between the agent’s estimate of the precision of the intended action and the sensory attenuation of the incoming sensory information (Clark, 2020). The role of precision is further emphasized by Kiverstein et al. (2024) on the account that affect plays a central role in the regulation of precision. This view makes use of the concept of affective charge as a regulator of action precision (Hesp et al., 2021; Smith et al., 2022). Because affect is felt in the body and thus scores its homeostatic dynamics, it links action precision to these dynamics and, consequently, first priors/preferences. In sum, this scheme makes a predicted action affectively desired in the context of the agent’s ecological affordances. These arguments raise the question whether the concept of desire is necessary to explain conation or can be subsumed under the affect-mediated precision of an action policy.

In this paper, we aim to further explore how AIF, as a theory of motivation, fits to conative behavior. We consider motivation as a hierarchy of inferences comprising the systems of minimal or preconative motivation and conation (Figs. 1 and 2). In turn, we present conation as a hierarchy of its own (Fig. 3). In this scheme, at the top of the conative hierarchy is what we call *raw desire* defined as a meta-preference (to exist as an idiosyncratic agent) and an inference on affective charge. As such, raw desire contextualizes subordinate affectively regulated desires and, further down the hierarchy, the minimal motivation (Fig. 3). We describe a putative neural architecture supporting this conative hierarchy (Fig. 1). We argue 1) that nested under raw desire, conation directly follows from the FEP, 2) that raw desire is isomorphic with the concept of drive and can explain generalized desires antecedent of environmental affordances, and 3) that the idiosyncratic hierarchy of conation can explain how agents in similar physiological states with the same first priors and ecological affordances would nevertheless choose different actions to realize their desires.

**Fig. 1:**
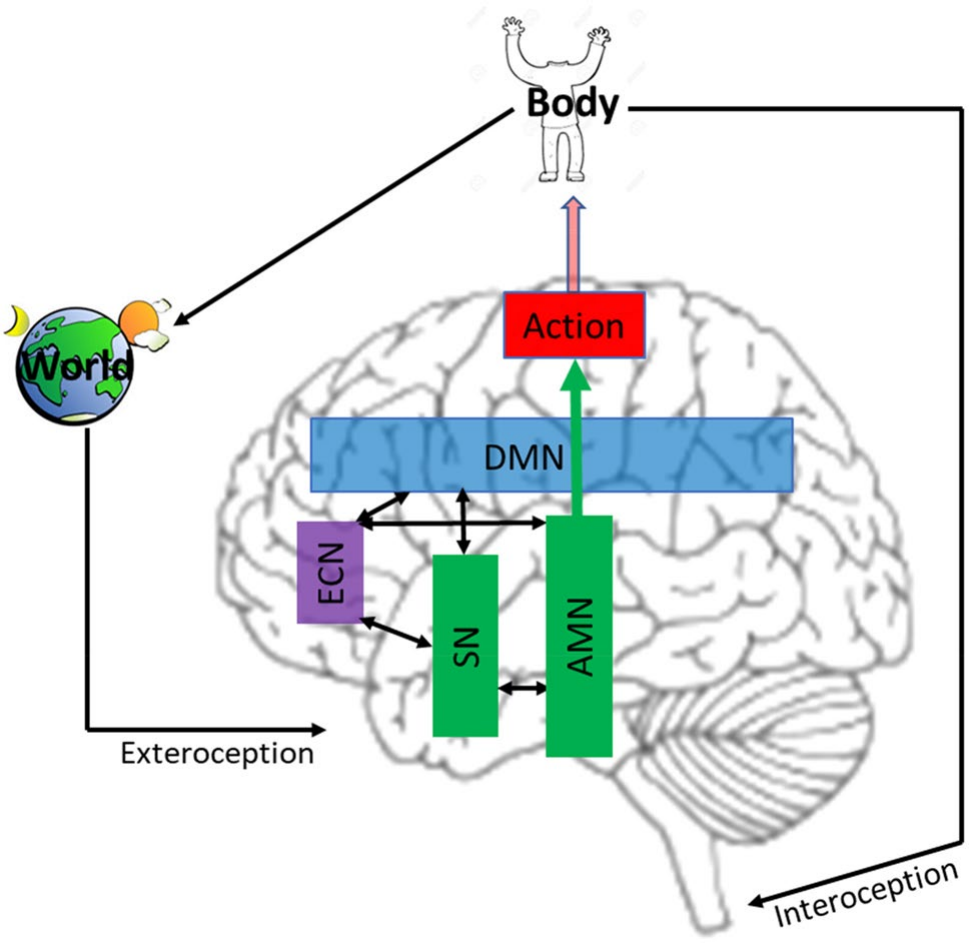
Structural network of conative behavior. The green represents the minimal motivation network; together with the blue and magenta it expands to the conation network. The arrows indicate the information flow. DMN =default mode network; ECN = executive control network; SN = the salience network; AMN = action mode network

**Fig. 2:**
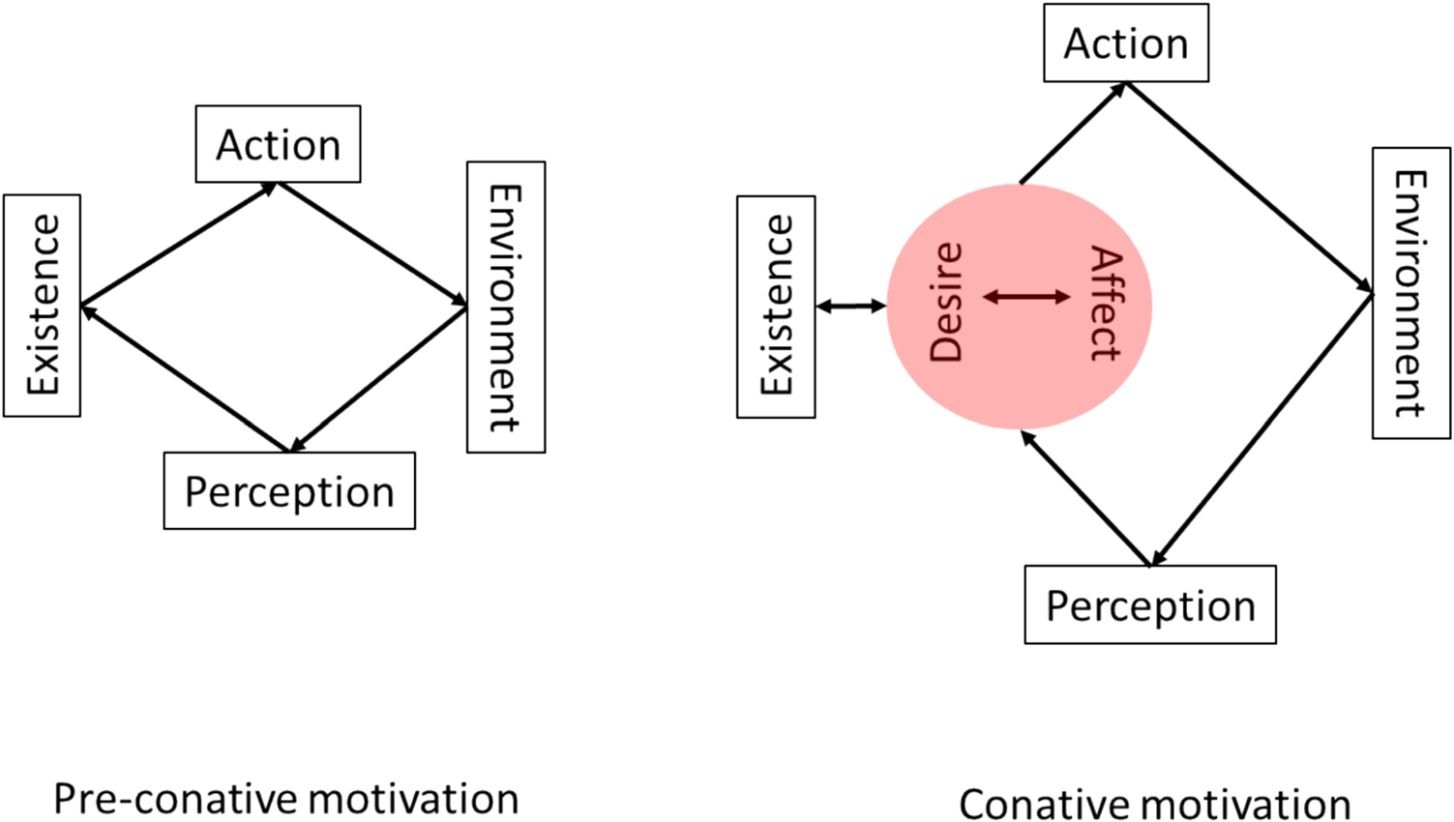
The difference between the minimal (preconative) and conative motivation. Arrows indicate the flow of information

**Fig. 3:**
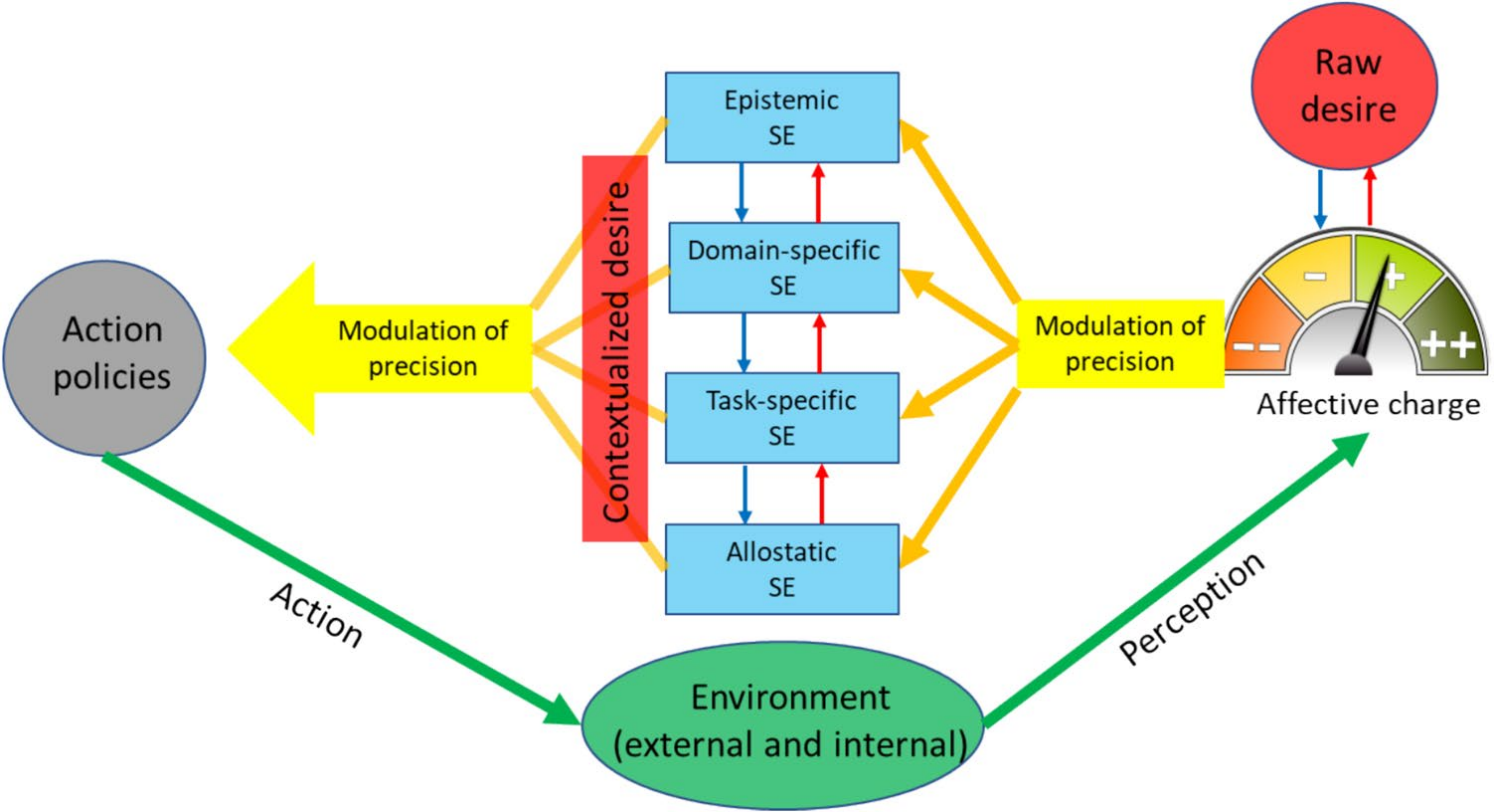
The functional network of desire. The blue and red arrows indicate the prediction-prediction error counterflow. The orange arrows indicate the modulation of precision. The green arrows indicate the exchange of information with the environment. SE = self-efficacy

We also propose that for better understanding of motivation, it is important not only to explain what drives behavior but also what drives inaction or *deferred action*, as we call it through the rest of the paper. It needs to be emphasized that deferred action, as understood here, is not just a delayed or postponed action but an undecided one that may or may not happen. We argue that according to the FEP, on which AIF is based, action is the default mode of a living organism, and that for highly developed organisms (mammals and arguably other vertebrates) being allostatic *and* autopoietic necessarily means being conative. Referring back to the above epigraph, we maintain that the real question about motivation is not why and how the pistol fires—this is what pistols are—but what takes it so long. We address how AIF may account for deferred action and, consequently, conation, with an emphasis on the role of affect. At the end, we consider clinical examples of distorted deferred action and desire.

## To be is to act

Active inference is a theory of sentient behavior developed over the past two decades and proposed as a “unified brain theory” (Friston, 2010). At its basis is the free-energy principle, which conditions the existence of self-organizing nonequilibrium systems on resisting the second law of thermodynamics^2^ and avoiding decay by minimizing their variational free energy (Friston, 2009). Such minimization is achieved by avoiding surprising states, that is, states uncharacteristic, and therefore unlikely, for a given phenotype. In this view, an organism embodies a generative model (GM) of itself and its environment, generating predictions about its states, in accord with its homeostatic set points, and states of the environment, whereas the actual states’ deviation from these predictions generates a prediction error. Increase in PE results in a greater surprise. Organisms and their environment are ever-changing entities, and their GM needs constant updating, which happens by comparing the model’s prior beliefs (priors) with incoming sensory input. Because surprise is usually computationally intractable, GM, instead, minimizes its variational free energy, which is a tractable quantity representing an upper bound on surprise. Thus, an organism can reduce its surprise and avoid decay by minimizing its GM’s variational free energy, which also means minimizing its prediction errors.

To be a GM of its environment, an organism must be conditionally independent of it, that is, separated by a Markov blanket. Otherwise, the organism will dissipate into the environment and cease to exist—a state of the highest surprise. In this way, the FEP is tautological with existence (Friston, 2012). Maintaining conditional independence of the ever-changing environment requires acting on it to regulate the exchange of matter and information, lest the organism succumb to the rising entropy and decay. This implies a hierarchy of first priors, where the initial prior is existence. The next down the hierarchy are priors on actions likely to safeguard the existence from the challenges of entropic decay, which can be viewed as tautological with the allostatic belief in “stability through change^3^” (McEwen, 2000) or, to paraphrase, *stability through action*. These are the foundational priors of self-organizing nonequilibrium systems. Whereas all organisms belong to this class of systems, only autopoietic ones qualify as organisms.

Evolution can only happen via autopoiesis (Varela et al., 1974). This principle is encoded in the structure of nuclear acids, the essential molecules of life, and is reified in the mechanisms of biogenesis. Accordingly, the third foundational prior of organisms is the autopoietic belief. Thus, from the free-energy and evolutionary principles it follows that the organism GM predicts its continuing existence and propagation through action (c.f., the notion of the brain as a self-evidencing GM; Hohwy, 2016). Therefore, all allostatic autopoietic agents are intrinsically motivated to exist, act, adapt, and reproduce. Consequently, we will argue that all motivation can be traced to these foundational priors and that they underwrite the mechanism of desire. A need to act seems like an obvious aspect of motivation; a more interesting one is the emergence in higher-developed organisms of the ability to defer and choose action.

## The natural history of deferred action

The simplest organisms such as bacteria are not known to defer action. They respond to environmental change with chemotaxis automatically. Their predictions about the concentration of extracellular chemicals translate directly into their movement (for an active inference account of bacterial chemotaxis, see Corcoran et al., 2020; Krupnik, 2024). Bacteria cannot defer action, because sensory signals from their receptors are directly transduced to their molecular motor (Wadhams & Armitage, 2004); therefore, their “perception” is not conditionally independent of their action. Bacterial chemotaxis is not a conative behavior (an automatic movement needs no desire), yet it provides for the robust adaptation of the most successful domain of life. This shows that although action is necessary for the existence of organisms, conation is optional and secondary to it.

The relationship between action and conation fits comfortably with the distinction between merely reflexive active inference and active inference as planning (Botvinick & Toussaint, 2012; Friston et al., 2025). Merely reflexive refers to the kind of generalized homeostasis that can be explained by acting upon the world to bring about predicted sensory consequences in the moment. This can be contrasted with active inference based upon inferred policies or plans that entail a generative model of the consequences of action. Since these consequences are in the future, they—and future actions upon which they are contingent—become random variables that have to be inferred. This introduces the capacity for conation in the sense that certain actions can be deferred. Mathematically, the move from merely reflexive inference to action selection based upon (Bayesian) beliefs about policies entails a particular kind of deep generative model, in which action per se is distal to internal belief updating and thereby becomes an inferred cause of sensory consequences (Friston et al., 2023). In short, systems implementing active inference as planning acquire an authentic kind of agency in the sense that they have the additional capacity to infer and defer their actions.

Conditional independence between perception and action appears later in evolution, where these functions are embodied in morphologically and physiologically distinct structures such as muscle-like contractible tissues and ganglion-like neural networks. Although these structures are found in rather primitive organisms, including worms and jellyfish, it is less clear when deferred action evolved. Nevertheless, insects already have it. For example, honeybees can watch the dance of a scout bee communicating foraging instructions and defer action until the dance’s end (Frisch, 1993). This example shows how deferred action can allow for higher-order cognitive processes, such as learning, memory, and planning.

AIF offers a straightforward explanation for the mechanism of deferred action. Action in AIF is the fulfillment of an agent’s prediction of being in a certain state, such that action completion is evidenced by a sensory sensation, e.g., proprioceptive. The prediction of raising the leg is confirmed by the proprioceptive feeling of the raised leg. This, however, is only possible if the proprioceptive sensation of the current state is transiently attenuated, because it presents a prediction error that would otherwise interfere with the prediction driving the movement (Brown et al., 2013). Because sensory attenuation facilitates the release of action, its absence offers a plausible mechanism of action deferral. More importantly, AIF provides a model—specifically, the deep temporal model (Friston et al., 2018)—that accounts for both action initiation and deferral. According to this formulation, the logarithm of the probability of an action (or action policy) is proportional to the negative of its expected free energy (EFE). expected free energy can be formulated as comprising two terms: the epistemic and pragmatic value of an action policy. These are often framed as the policies of exploration and exploitation, respectively. Notably, exploration can occur through purely mental actions, with physical actions deferred. In the above example of a bee dance, deferring an overt physical action while learning the foraging instructions allows for epistemic exploration, which is then followed by the act of exploitation through physical foraging. In the epigraph to the paper, deferring the pistol shot to the second act allows for contextual exploration of the likely outcome. Accordingly, we can specify a particular case of EFE formulation as comprising epistemic exploration via action deferral and pragmatic exploitation via action release.

Sensory attenuation may play an essential role in the cycle of epistemic exploration and pragmatic exploitation. Under active inference of a nonreflexive kind, action sequences are selected by their ability to minimize EFE or expected surprise. Expected surprise can be reduced in two ways. Because expected surprise is uncertainty, one way is to indulge in epistemic, exploratory behaviors, which are intrinsically motivated. The other way to minimize expected surprise is by avoiding outcomes that would be surprising for the agent in question. This extrinsic motivation is determined by pragmatic imperatives based upon the agent’s prior preferences, i.e., beliefs about outcomes that are typical of it. In AIF, these motivations are understood as prior beliefs about action policies, and these policies are realized through merely reflexive mechanisms.

To realize the intended consequences of action, it is necessary to suspend attention to sensory (e.g., proprioceptive in case of motor action) sensations supplying evidence that one is not acting. This sensory attenuation can be regarded as the complement of selective attention and rests upon the attenuation of sensory precision (the precision of sensory input). Note that selective attention and sensory attenuation are covert actions that enable the merely reflexive mechanism that engenders overt action. In this setting, the precision of beliefs about policies (i.e., policy precision) can have a profound effect on policy selection and ensuing action, both covert and overt. Agents, in whom policy precision is itself inferred, have a special kind of self-evidencing based upon (Bayesian) beliefs about agency and motivation that are elaborated later with regard to self-efficacy and affect.

It needs to be clarified that the partition into exploration and exploitation does not have to lie between the epistemic and overtly physical domains. Overt behavior can be exploratory, e.g., roaming for a prey, and exploitative, e.g., chasing it. Likewise, purely mental acts can be partitioned into explorative such as epistemic foraging, e.g., using heuristics to figure out the best hunting grounds, and exploitative such as epistemic consumption, e.g., computing the best trajectory for a chase. Such a partition is determined by the hierarchy of free-energy minimization processes, where exploration supervenes on exploitation. Sensory attenuation-like mechanisms can operate at different levels of cognition form proprioceptive (for motor movement) to highly abstract like playing chess. To map out a series of moves and counter-moves, a chess player must be able to “attenuate” his attention on a current move to plan the next one.

Arguably, the main criticism of the AIF account of motivation is that motivation is not just computed but felt as value-laden, which is encoded even linguistically in the common phrase “I *feel* like doing such and such.” This criticism considers desire as an essentially affective state. Bellow, we frame desire and motivation in structure functional terms to suggest that AIF accommodates their affective nature.

## Desire for what and what for?

Desire is commonly viewed as a mindset that drives the agent toward a goal state, something which is desired, in other words, a drive or “motivational force” (Colombo, 2016; Klein, 2018; Yon et al., 2020). It is contrasted with a belief state, which represents the world’s regularities and supposedly has no motivational force. To take a closer look at these presuppositions, we first ask what an agent is driven to, that is, what it desires. The intuitive answer is that it desires something of value either in the world around, such as a candy, or within, such as a good feeling. This, however, is a false dichotomy, because the brain has no direct access to the outside world, and hence, its only object of desire is its internal goal states. According to the free-energy principle, the brain’s goal state is its minimal free energy (Friston, 2010). Because the brain can only exist as part of a body, minimization of its—or more precisely its generative model’s—free energy is predicated on the agent’s being in its phenotype-typical states. These states comprise the agent’s “first priors” (Allen & Tsakiris, 2019) and are, therefore, predicted preferred states, to which all other states are subordinate. This allows AIF to equate desire with prediction and account for value by parametrizing it as preference (Smith et al., 2022).

Even if a predicted state has a value and is thus desired, this still does not answer the question of why desire needs to be experienced/felt to be acted upon. To pursue its preferred/desired states, it is sufficient for an agent to just predict them and select an action policy with the minimal expected free energy. Habitual or automatic behaviors can achieve goal states without desire (Schwartenbeck, FitzGerald, Mathys, Wurst et al., 2015b). Indeed, many if not most actions are taken without desire and awareness. To clarify the distinction we make between motivation and desire, here, motivation is viewed as underwriting any behavior, whereas we reserve desire for the conative motivation underwriting deliberate, affect-laden and conscious decision-making (Desire ⊇ Motivation). In this view, the minimal motivational network (Fig. 1 in green) requires no desire to effect habitual or automatic behavior, which can be mediated by low level subcortical processes sufficient for merely reflexive affective inference. Conation may require a much deeper GM of the consequences of inferred and deferred action. Such a model is likely supported by a larger network (Fig. 1) that in addition to subcortical regions includes cortical networks such as the executive control network (ECN), the salience network (SN), and the default mode network (DMN). These are known as intrinsic networks, with SN responsible for recruiting attention and processing of emotionally salient stimuli; ECN responsible for action selection and planning and, consequently, for emotion and impulse control; DMN responsible for recruiting attention to the self and integrating and evaluating self-relevant information over long-term timescales that includes past memories and future counterfactuals (Raichle, 2015). The DMN is active during “resting state,” when attention is not directed outward, and the agent is not engaged in any task. It is believed that the SN functions as a switch, turning attention outward and activating the ECN to engage the task at hand. Accordingly, the DMN is known to activate in counterphase with the SN and ECN. This scheme is known as the triple-network hypothesis (Menon, 2019). More recently, however, another intrinsic network, the action mode network (AMN), has been identified (Dosenbach et al., 2025).

The AMN is responsible, as its name implies, for turning on and sustaining the organism in action mode, including increased arousal, sustaining an action-congruent physiological state, goal maintenance and action planning, and viscerosensation. In the authors’ words:

The AMN initiates, maintains, and controls a domain-general action-mode of brain function in which many specific behaviors may be performed, from complex mental tasks to physical actions, wherein AMN regions implement all the shared common processes these actions require (Dosenbach et al., 2025).

Both DMN and AMN are widely distributed networks; DMN is spread out from the prefrontal through posterior cingulate cortices and AMN—from the brain stem through the supplemental motor area. In their activity, AMN is maximally offset from DMN. This structure-functional architecture partitions the brain activity into two domain-general cognitive modes:

Self-referential and action-oriented, driven by DMN and AMN, respectively, with SN and ECN contextualizing them to the task and situation at hand (Fig. 1).

Considering action as the brain’s primary function and AMN’s role in its planning, initiating, and sustaining, the implied role of DMN is to compute action’s (or inaction’s) value for the organism over different timescales. From the AIF standpoint, that (Bayesian) value equals the decrease in the expected free energy. In other words, desire, in its most general sense, can be read as a DMN’s prediction of the organism’s state *other* (with a lower free energy) than the current one, which also can be seen as a meta-preference. Then, AMN’s function is to plan and execute a policy likely to bring that state about. This scheme places DMN at the top of the conative hierarchy. This also means that desire comes later in evolution with the development of DMN.

To estimate the value (EFE) of planned action or inaction over large timescales, the DMN would need to infer it based on some generalized dynamic parameter. Affective charge has been proposed as such a parameter, which is a domain-general property of affect that scores the GM’s error dynamics, where accelerating and decelerating rates of prediction error minimization are associated with, respectively, positive and negative valence (Hesp et al., 2021). By extension, affective charge scores the precision or confidence associated with the evaluation of policies. This precision is inversely related to the expected free energy averaged over policies. As such, affective charge reflects the agent’s “subjective fitness” in the sense of the agent’s subjective evaluation of the adaptive value of its behavior. There is empirical and modelling evidence (Hesp et al., 2021; Schwartenbeck, FitzGerald, Mathys, Dolan, Friston et al., 2015a), suggesting that phasic dopamine discharge encodes fluctuations in policy precision, which can be read as affective charge dynamics. Heuristically, affective charge reports the agent’s confidence about its future brought about by prospective policies. This makes an increasing affective charge the default prior, which can be regarded as a higher-order first prior. Simply put, any feeling agent not only expects to exist, but to feel good about making its existence, or in other words, it *feels like acting* in a way that evidences its existence. This adds another layer to the minimal motivation, where it is supported by the temporally deep self-conscious circuit of affective desire (Fig. 2). An obvious advantage of such a design is a more robust existence, which would be selected for in evolution.

In this scheme, conation/desire^4^ is a hierarchy of inferences (Fig. 3). At the top, it starts with *raw desire*, a domain-general inference on the dynamics of the agent’s affect. This inference is mediated by affective charge, making it a higher, third-order inference, because affective charge is itself an inference on prediction error dynamics. Simply put, raw desire is a *conscious* expectation to feel better, which is realized down the hierarchy by corresponding action policies. This is similar to a recently proposed affective gradient hypothesis (Shenhav, 2024), where affect is considered as a hierarchically highest source of motivation (although in a more nuanced way, where an agent may want to feel better along different affective dimensions, i.e., arousal and valence). To that, we add the layer of raw desire as an explicit inference on affective dynamics that integrates arousal and valence in a “feel better” vector. As such, raw desire can be seen as the affective aspect of consciousness, that is, being conscious of one’s affective dynamics (affective charge).^5^ In this scheme, the conscious nature of desire follows from the view of affect as an elemental form of consciousness (Solms & Friston, 2018). The putative function of affective charge is modulation of the precision of action policies, where a higher affective charge renders the policy more precise (Hesp et al., 2021). In so doing, affective charge becomes contextualized by the corresponding action policies. This is the step in the hierarchy of desire, where the raw desire develops into a *contextualized desire* (Fig. 3). It has been proposed that such contextualization is mediated by the hierarchical system of self-efficacy, which spans the gamut from epistemic through allostatic self-efficacy (Krupnik, 2024). The proposed model of conation makes it an autoregulatory closed loop (Fig. 3), starting from raw desire as a prior on affective charge and receiving, via affective charge dynamics, PE from the outcomes of action policies regulated by the affective charge.

Framing desire as the process of inference on affective charge makes it affect-dependent and conscious. It also adds an interesting twist: at its most abstract level, desire is not about affordance-dependent preferred states but is a meta-preference, to which ecological preferences are subordinate. Thus, my *raw* desire for a candy is not about the candy but about *me* feeling better. This can explain how some behaviors, once overlearned, can become desire-independent and intrinsically motivating. If we remove desire from the conation loop (Figs. 2 and 3), it becomes truncated to the minimal motivation, bypassing affect and desire. This way, a behavior, once desired, may “run away” by self-perpetuating as predicted with high precision and thus develop into a habit as proposed in (Schwartenbeck, FitzGerald, Mathys, Wurst et al., 2015b). Habit-formation has implications for psychopathology discussed later in the paper. The suggested conceptualization of conative motivation transcends the belief-desire dichotomy by making desire a belief about affect and, at a lower level of the hierarchy, goal states.

The model offers an extended perspective on conation by framing it as a process of self-modeling and self-evidencing, as does the aforementioned affective gradient hypothesis (Shenhav, 2024). An agent inferring the precision of policies must have a second-order inference, i.e., affective charge or subjective fitness, about that precision. This inference (about the precision of a policy) is contextualized by and conditioned on the agent’s state of mind, which then can be viewed as intentional and affective and must itself be inferred (a third-order inference) to contextualize the precision of that policy. Such a hierarchal Bayesian inferential process is in line with the AIF account of self-modeling and phenomenology (Limanowski & Friston, 2018; Sandved-Smith et al., 2021). In this scheme, conation follows directly from the FEP. If we take its self-evidencing reification (Hohwy, 2016), we can say that agents with the minimal motivation network must self-evidence (maximize Bayesian model evidence of their GM) to exist, whereas conative agents must also *desire* (a higher-order first prior) to self-evidence, that is, to *feel like* evidencing their existence in the form and shape they are. To illustrate by using Clark’s example (Clark, 2020), I want/desire to go watch a movie, because I feel like evidencing my existence as a creature that I am, i.e., one that enjoys going to the movies, which can be seen as another tautological reification of the FEP. This makes both existence and self-evidencing intrinsically desirable, which can explain the intuitively paradoxical fear of death and aversion to boredom. The fear of death may be considered paradoxical, because, strictly speaking, death cannot be feared; it is an experientially empty state that by itself harbors no danger. What may be scary about being dead is the loss of the pleasure of existence (a more in-depth account of this subject can be found elsewhere; Krupnik, 2025a). The aversion to boredom appears paradoxical, because the state of mental and physical idleness is not in any way harmful. From the standpoint of raw desire, however, it deprives the agent of the pleasure of self-evidencing (via mental or physical action).

Note, that my high-order desire for entertainment is linked to my homeostatic needs via affect and interoception, thus spanning the whole hierarchy of conation from conscious to unconscious and closing the conative autoregulatory loop, which also means that preaffective systems/organisms are also preconative (Fig. 2). The additional layer of conation, as we argue below, allows for a more flexible consciousness-mediated behavior without the agent being overwhelmed by the complexity of life-demands and choices, while also opening the door for psychopathology, where that flexibility is compromised,

The proposed hierarchy of conation allows for generalized desires that are subordinate to raw desire but antecedent to environmental affordances. For example, generalized epistemic desire for knowledge and certainty (curiosity) regardless of particular epistemic affordances. Such a desire was modelled by Smith et al. (2022) as the epistemic value term in the expected free energy equation. Likewise, one can think of the desire for fairness as independent of the context, for entertainment regardless of the kind, or for attachment, happiness and well-being across different timescales and situations.^6^ Raw desire complements first homeostatic priors (Allen & Tsakiris, 2018) in that homeostatic predictions are context-bound, relatively short-term, do not require consciousness, and could be satisfied by the minimal motivation network (Figs. 1 and 2). Extending first priors to broadly understood phenotype-typical states as suggested by Smith et al. (2022) may not cover generalized desires, because they also include learned idiosyncratic beliefs or empirical priors (and their precision) about what kind of agent one is. Thus, raw desire may be seen as the first prior, that is, a desire to exist in the personal trait-specific way, to which all other wishes can be traced. This view recapitulates the FEP, where all behavior can be traced to the imperative of reducing free energy. It also helps understand how and why physiologically and genetically similar organisms manifest diverse and flexible behaviors under the same circumstances. Even monozygotic twins display inter-individual behavioral differences within similar settings (von Stumm & Plomin, 2018), which may speak to the role of empirical priors in shaping personhood, including its conative aspects. To sum the whole argument up, the proposed model of hierarchical conation allows us to answer Clark’s question (Clark, 2020) about where desire comes from. It comes from the agent’s top-down inference about how it *expects/wants* to be, which is continually updated from bottom up by its environment (via active and perceptual inference) about how it *can* be. As an aside, the outlined hierarchy of desire is consistent with the notion of the primacy of drives in the drive theory, especially its late revision, where drives are considered conscious (Solms, 2021).

The existence of certain conative traits and generalized conditions, such as impulsivity and anhedonia, respectively, appears consistent with the notion of metacognitive raw desire. Even though both impulsivity and anhedonia may be expected to reside within the reward-processing circuitry, DMN has been shown to play a role in both. For example, resting-state DMN activity predicts impulsivity or trait-urgency (Gentili et al., 2020; Zhao et al., 2017). Likewise, resting-state DMN-SN functional connectivity correlates with anhedonia scores (Imperatori et al., 2025), and an anhedonia-specific hypoactivation of resting-state DMN was found in children with anhedonia (Pornpattananangkul et al., 2019). Importantly, deep brain stimulation of the anterior DMN, specifically, the ventromedial prefrontal cortex (vmPFC), can reverse the anhedonia-like behavior in an animal model of depression (Hamani et al., 2012), and stimulation of the medial forebrain bundle in depressed people has an anti-anhedonic effect (Boscutti et al., 2025) presumably via the activation of the descending vmPFC projections (Fenoy et al., 2022). The involvement of DMN in generalized aspects of desire can be seen as a higher-order inference in the context of the self-model. We can speculate that the aforementioned alternating activation of DMN and AMN reflects the switch between the self-referential and outward ecological desire, respectively. Thus, following the view of psychopathology as “optimal inference with suboptimal models” (Schwartenbeck, FitzGerald, Mathys, Wurst et al., 2015b), we may specify a subset of such suboptimal models as ones with inflexible precision of desire. It is important to acknowledge that DMN’s involvement in trait-like aspects of desire is insufficient to conflate its activity with raw desire. What is missing is a temporal link, where DMN activation precedes a goal-directed desire and an associated dopamine discharge in the midbrain. No such data are available, to our knowledge, although a brain imaging study to test this hypothesis seems plausible.

Turning to the second half of this section’s title, we need to explain the putative functional advantage (besides the aforementioned evolutionary advantage) of adding desire to the minimal motivation network. Several such advantages are apparent. One concerns the limitations of the minimal network noted by the critics of the purely doxastic view of motivation. Given the multitude of environmental demands in multiple domains of the state space, it appears that the brain GM can be easily overwhelmed unless it prioritizes a small subset of goal states (Klein, 2018). An average person must act on many goals at different hierarchical levels at the same time: driving, planning a meeting, answering (or not) a phone call, negotiating the homeostatic needs, etc. Attending to each domain may seem straightforward, but prioritizing and reconciling these priorities in the dynamic world may be computationally challenging. Desire, by virtue of being a hierarchical autoregulatory process that is domain-general at its highest level, can dynamically optimize the goal state priorities based on their relative affective salience. Moreover, as a conscious meta-preference, raw desire can exert flexible control over subordinate desires in the context the agent’s GM (including the self-model) over different timescales. Such temporal depth is another advantage, because it allows to make inferences on affective charge over large time scales both in the past and future, e.g., one can infer an increased affective charge at the memory of the last night’s party or a decreased affective charge on the account of one’s own mortality. Therefore, the mortality is not just predicted with a high probability but experienced as undesirable, and this conflict provides an opportunity for a more elaborate cognitive processing. Temporal depth is not particular to desire; it is a property of the brain GM (Friston et al., 2018). However, desire renders it conative.

A third advantage is the dynamic deconfliction of motivation. This is different from the discussed above prioritization in that conflicting goals (predicted states) can arise within the same domain. Such deconfliction is possible owing to the proposed hierarchical structure of motivation, where the raw desire is linked to goal states *indirectly* through affective charge (Fig. 3). Specifically, a preferred affective state, which by default (as a first prior) is an increased affective charge, may be in conflict with the preferred state. Before illustrating this supposition with an example, some general considerations are in order. The ultimate function of the brain GM is the minimization of the free energy (Friston, 2010) and not necessarily making the agent feel good. This can be paraphrased as a conflict between making an agent feel good and making sense. Also, whereas an increased affective charge is the default desire, it cannot be indefinite. Free energy cannot be zero; therefore, an affective charge cannot grow indefinitely, which means that the desire for (prediction of) its increase will inevitably come to a contradiction with the free energy dynamics. This means that desire is bound to oscillate between inferring positive and negative affective trends.

How could an agent desire (predict) a decreased affective charge, given that it scores the agent’s subjective fitness (Hesp et al., 2021)? That would mean wanting to fail. Nevertheless, we argue that the answer is in the hierarchical structure and dynamics of motivation. Let us take the frequently invoked in the literature example of hunger. A drop in your glucose level signals an allostatic load and generates a PE at the allostatic level. The PE is passed up the cognitive hierarchy and is indexed by a decrease of the affective charge, which generates a PE at the level of desire (Fig. 3), indicating that your GM needs updating. This, as noted above, can be accomplished by either perceptual or active inference. Engaging active inference, you decrease your expected free energy by deciding to look in the fridge but find it empty. At this point, your GM has to select a policy on a longer timescale to reduce the EFE (e.g., to go to the store or skip breakfast and wait until lunchtime). Until that policy is executed, however, your hunger grows, and the GM’s current free energy is increasing. The only option to reduce it in the near term is perceptual inference, i.e., updating the GM at the level of raw desire. This would mean predicting/desiring a transient drop in the affective charge (in psychological terms, known as distress tolerance). As an old adage has it, “things will get worse before they get better.”

While trivial, this example has significant ramifications, because *counter-hedonic inference* can be vitally important. Consider a predator that operates on a thin energy margin and has to compute whether to conserve its energy while tolerating hunger a little longer or to risk wasting it by lunging at the prey under suboptimal conditions. Of note, lower distress tolerance is associated with the trait impulsivity (Borges et al., 2017).

One can also think of a counterexample of *hedonic inference* serving the deconfliction function. In the above hunger case, the agent can use an alternative perceptual inference strategy by increasing the precision of the hedonic prediction thus discounting the hunger PE and temporarily “ignoring” it, until the environment provides a suitable affordance (e.g., a café on the way to work). Although both strategies have the same immediate effect, their long-term consequences are different. The counter-hedonic strategy locks the agent in a “depressive” proposition conditioned on the future implementation of a delayed but decided upon policy, such as waiting to eat until lunchtime. The hedonic strategy, on the contrary, leaves the agent more flexible and open to exploit ecological contingencies, such as the aforementioned café or a snack vendor. Such contradictory strategies are related to a more general model-antimodel Bayesian dialectic explored elsewhere (Krupnik, 2025a, b).

The deconfliction function of desire may be especially important in high complexity, ambiguous social settings with shifting loyalties and alliances. Malfunction of desire, on the other hand, may play a role in psychopathology, as discussed later in the paper. It is tempting to speculate that the emergence of conative motivation was one of the evolutionary reasons for the development of DMN, which would suggest that simpler, pre-vertebrate organisms that lack DMN have no conation, only the minimal motivation.

## Deferred action aids the maturation of desire

In active inference, motivation is approached from a doxastic standpoint that can be expressed as “I do what I believe.” The belief is that a certain action will reduce the agent’s expected free energy, which is a necessary condition for its existence in a phenotype-typical state. Then, an action that is most likely to do so is executed. This explanation suffices for motivation of simple homeostatic agents such as bacteria, in which homeo- or allostatic action is automatically triggered by survival and autopoietic needs, but it may appear unsatisfactory for higher-developed organisms with a brain. Such organisms have to navigate a vast and rich counterfactual belief space (Wilkinson et al., 2017), where actions are often far removed from their survival consequences and are not automatic but require elaborate planning and decision-making. Returning to the hunger example, where I contemplate getting food out of the refrigerator, selecting to go for it would satisfy the minimal EFE imperative, unless I decide to do nothing and stay hungry based on a larger set of predicted goal states (e.g., other time commitments, diet, etc.). Later in this section, we discuss conditions that release action, but now focus on what it takes to get me moving. To do so, my action not only has to be the most likely in the current context (having the lowest EFE), but I need to want, *feel like*, doing it. In other words, on a phenomenological level, I need to have a desire. Note that this implies having both a desire for the object and state (food and satiety) as well as for the action (foraging).

In a recent AIF development, desire and affect have been factored in the EFE equation (Hesp et al., 2021; Smith et al., 2022). In short, the argument is as follows. The EFE equation can be written as comprising the risk and ambiguity terms (referred to in the previous section as pragmatic and epistemic gain, respectively). The risk term scores the probability of the agent to find/observe itself in a preferred state, e.g., satiety, as the outcome of an action. This term then can be seen as scoring the desire for a state, as it equates the belief about the consequence of an action with the preference for a state, thus conferring the value property on the belief.

The same logic can be applied to the desire for an action. The probability of an action also depends on the agent’s confidence that it will bring about the expected outcome. Confidence is understood here not in a subpersonal sense as encoded in the precision (of an action policy) parameter. That precision, as noted in the previous section, depends on the agent’s affect, more specifically, affective charge (Fig. 3). This explains how the desire to act is both a belief and a feeling. Thus, the EFE equation accounts for desire’s doxastic, value, and affective aspects. Next, we build on the AIF theory of desire to highlight the role of deferred action.

Any action implies inaction (e.g., the aforementioned model-antimodel dialectic), which dialectic is transcended by motivation and decision-making. Returning to the above example, we need to explain why would I not simply adjust my GM to expect feeling hungry, sit back, and starve to death (an AIF account of self-destruction has been suggested before Krupnik & Danilova, 2024).^7^ The answer is straightforward for actions directly related to homeostatic needs, because inaction leads to a state of high surprise/EFE and thus feels deeply unpleasant and undesirable. It may not, however, be so obvious in low-stake decisions, e.g., when trying to rest, to go or not to go on social media? To better understand such choices, we need to compare the *experience* of action versus deferred action, because the answer mainly depends on what an agent *feels like* doing.

Considering the pragmatic and epistemic gains in the EFE equation, the probability of an action depends on a trade-off between them (Schwartenbeck et al., 2013), meaning that the agent acts to explore or exploit whichever results in the lowest EFE. Deferred action, on the contrary, depends exclusively on the epistemic gain (here, we define deferred action as contrary to overt physical action, because mental action, strictly speaking, never stops). The main argument of this paper, therefore, is that deferred action affords the agent an opportunity for epistemic exploration and contextualization of its raw desire (Fig. 3).

To develop this argument, we refer to the AIF theory of motor control, specifically, sensory attenuation (Brown et al., 2013). Sensory attenuation, as discussed earlier, is necessary for a motor movement that would otherwise be inhibited by the prediction error generated by the sensation of the current body state. This also entails that the agent cannot be conscious of the movement’s sensations during it, but only before and after, because it is impossible to be conscious of an unfelt sensation. Such awareness would result in a series of stop-and-go jerks instead of a smooth trajectory. We suggest that such transient suspension of consciousness may apply, at a higher level, to decision-making as well, where the agent can be conscious of its decision to act before and after the action but not during. If so, the function of deferred action may be to allow motivation to mature beyond the minimal network to the level of conscious desire and the default mode network. This is consistent with the fact that habitual behavior is often automatic, that is, initiated unconsciously without a desire.

Time afforded by deferred action may be required to integrate affect, which is felt viscerally and slowly relative to the cortical processing, into the estimate of the EFE of a planned action. The dynamics of the affective charge during deferred action will determine the planned action’s desirability and, therefore, its precision and likelihood. These hypothesized dynamics are the meaning of the notion of “feeling like doing [something].”

On the neural level, the dialectic between action and inaction may be reflected in the interaction between the brain intrinsic networks. The executive control and salience networks are believed to be responsible for precision estimation and action selection (Schwartenbeck, FitzGerald, Mathys, Dolan, Friston et al., 2015a), whereas the default mode network is for self-referential and context-specific cognition (Raichle, 2015). Accordingly, we would expect upregulation of DMN and downregulation of ECN and AMN during deferred action and the reverse dynamics at its initiation, which could be tested experimentally as in Sridharan et al. (2008). In fact, trait and age-related impulsivity correlates with increased functional connectivity between DMN and the dorsolateral premotor cortex (part of AMN) responsible for action planning (Shannon et al., 2011), which can be interpreted as a deficiency in the deferred action’s function. There are more conspicuous examples of psychopathology that can be viewed as disturbances of deferred action.

## Malfunction of deferred action and desire

A substantial and growing literature frames psychopathology in the active inference terms, where a central idea is that psychiatric disorders manifest aberrant precision estimates of the agents’ models of the world, themselves, and their actions (Smith et al., 2021). A review of this literature is outside this paper’s scope. Instead, we focus on the role that deferred action and desire play in these models. There can be two kinds of impairment of deferred action: hyper- and hypofunction. Obsessive-compulsive disorder (OCD) is an example of the latter. A core symptom of this disorder is voluntary compulsive actions that are associated with negative affect (*Diagnostic and Statistical Manual of Mental Disorder,* 5th ed. text rev; *DSM-5*; American Psychiatric Association, 2022). Obsessive-compulsive disorder has been identified as a condition contradicting the active inference account of motivation, because compulsive actions, while predicted, are undesired, which invokes the belief–desire dichotomy. AIF accounts of OCD have been proposed before (Fradkin et al., 2020; Kiverstein et al., 2019; Levy, 2018), but we offer an alternative interpretation as it relates to motivation and desire. For simplicity, we will use the example of compulsive handwashing to explain it as impaired deferred action and to argue that it is consistent with the AIF view of desire.

From the AIF standpoint, handwashing at the onset of OCD is driven by the prediction of the preferred state of the decontaminated hands, which makes this state both desired and predicted. The precision of this policy is expected to increase with time, because it is associated with a transient relief from distress about the perceived contamination (Salkovskis, 1999) and, therefore, with a positive affective charge. A central question to answer is why everyone does not compulsively wash their hands, because nobody wants them contaminated, especially because this policy is socially encouraged, which is likely to increase its precision even further. Our hypothesis is that OCD patients fail in deferred action. Deficiency in deferred action prevents an adequate context-specific processing of the alternative inaction policy and thus deprives OCD patients of choice. A possible mechanism of this deficiency is hyperactive sensory attenuation, where patients attenuate their sense of contaminated hands and initiate handwashing to be in the predicted decontaminated state. This kind of hyperactive sensory attenuation can be regarded as the complement of a failure of sensory attenuation that can lead to impaired initiation of action in psychiatric and neurological conditions (e.g., schizophrenia (Adams et al., 2013), delusions and Parkinsonism (Brown et al., 2013), stuttering (Usler, 2025)). In addition, OCD patients have low distress tolerance (Laposa et al., 2015) and may be unlikely to enact a counter-hedonic inference. If so, they would instead, as discussed in the previous section, rely on a hyperprecise hedonic prior (no distress) and seek environmental affordances, of which there are plenty for handwashing. As OCD progresses, and the compulsion cycle gets overlearned, the behavior may become “habitisized,” i.e., intrinsically motivated (Smith et al., 2022). At this stage, the compulsion becomes predicted with a high precision as both an expected state and an action policy, which compounds the challenge of controlling the compulsion despite the person’s conscious desire and effort.

Consistent with this idea, neurological data show that OCD patients fail to coordinate the activity of the default mode and executive networks. Their DMN fails to downregulate during externally focused activity (similarly to the above-mentioned impulsive subjects) and instead remains constitutively hyperactive (Gonçalves et al., 2017). Moreover, deep brain stimulation of the ventral striatum was shown to restore normal DMN-striatum functional connectivity in OCD patients and decrease their symptoms (Figee et al., 2013). Of note, the progression to habitual compulsion in OCD parallels the well-studied progression from impulsivity to compulsivity in addiction (Dalley et al., 2011; Lee et al., 2019).

Interestingly, a deficiency in another kind, that is, interoceptive sensory attenuation has been implicated in OCD. Hypoactivity of interoceptive sensory attenuation was suggested as a mechanism of compulsion, where the inability to attenuate interoceptive prediction errors results in the sense of incomplete action (Roberts et al., 2021). Such failure of sensory inhibition has been demonstrated in OCD patients (Gentsch et al., 2012). This combination of hyperactive exteroceptive and hypoactive interoceptive sensory inhibition can lock OCD GM in the vicious cycle of a compulsive wish/prediction to act without the ability to experience the intended outcome. In this case, the model would continue predicting but failing to decrease the expected free energy via the intended action. Below, we present depression and anhedonia as a mirror image of OCD in terms of deferred action’s malfunction.

Depression can be seen as a hyperfunctioning deferral of action resulting from hypoactive exteroceptive sensory attenuation. A lack of desire and pleasure are among criteria for major depressive disorder (DSM-5-TR). Indecisiveness and rumination have also been associated with depression (Hallenbeck et al., 2022; Nolen-Hoeksema et al., 2008). Taken together, these symptoms appear to describe a deferred action syndrome, where desire is arrested at the level of raw desire failing to contextualize into an action policy (Fig. 3). The cognitive meaning of depression can be interpreted as a belief that no action can plausibly result in the agent’s preferred states/observations (Stephan et al., 2016; Gilbert et al., 2022; Krupnik, 2021, 2024), which is also the AIF meaning of hopelessness (Krupnik & Danilova, 2024), another symptom of depression. Therefore, any action policy would have a low precision and be difficult to commit to, which can explain the indecision. The negative affective charge associated with the agent’s inability to decrease the expected free energy (perceived as a low subjective fitness) will further depress the agent’s desire in acting, which in severe depression, can reach the level of catatonia (Solmi et al., 2018). Both negative affectivity and low precision can be mediated by decreased dopamine transmission (Hesp et al., 2021), which is especially prominent in severe depression (Dunlop & Nemeroff, 2007). The hypothesized function of deferred action, as discussed earlier, is a comprehensive context- and affect-dependent estimation of the precision of an action policy. This explains rumination as a core feature of depression (Nolen-Hoeksema et al., 2008) and is supported by the observation that depressed people fail to disengage their DMN and to fully engage the executive network when involved in externally focused cognition (Belleau et al., 2015).

One proposed role for rumination, or “analytical rumination,” is an adaptive problem-solving meant to help the depressed find a solution to their predicament (Andrews & Thompson, 2009). In a different, AIF-inspired, point of view, rumination is a means of self-evidencing by a depressive generative model (Krupnik, 2024) that simulates/resamples negative outcomes as evidence of its failure. In support of this idea, rumination has been shown to associate with negative affect (Nolen-Hoeksema et al., 2008). The self-evidence hypothesis aligns with the view of psychopathology as “optimal inference with suboptimal models” (Schwartenbeck et al., 2015b). The depressive GM can be seen as an adaptation to allostatic overload—estimated as insurmountable—by predicting and fulfilling the GM’s failure in order to reduce its EFE.^8^ In our model, such a strategy would rely on counter-hedonic inference with the ensuing discounting of environmental affordances. Indeed, depression has been linked to a deficit of reward-seeking approach behavior in humans and model animals (Alcaro & Panksepp, 2011; Bakker et al., 2017, 2018). Rumination has also been viewed as a failure of sensory attenuation that results in the person’s inability to initiate action due to focusing on the current depressed state and thus overweighting the prediction error (Badcock et al., 2017). This is consistent with depression seen as deferred action’s hyperfunction due to a hypoactive sensory attenuation.

On the interoceptive side, anhedonia can be viewed as hyperactive sensory attenuation, a kind of “selective [hedonic] interoceptive blindness,” where hedonic prediction errors are underweighted (Krupnik, 2020). Consistent with this idea, there is ample evidence for the decreased activation of the salience network during reward processing in depressed people (Höflich et al., 2019; Kieslich et al., 2022). At the same time, higher activation of the ventral striatum in response to reward PE was found to associate with less anhedonia (Eckstrand et al., 2019), and deep brain stimulation of the medial forebrain bundle (the hub of reward and hedonic processing) had a hedonic effect in treatment-resistant depression (Boscutti et al., 2025). The discounting of hedonic PE would provide evidence for the counter-hedonic GM, leading to the reduction of free energy, which could explain the relative stability and often chronic course of depression. Thus, the vicious cycle of anhedonia, contrary to OCD, is wishing/predicting inaction to confirm the higher-order counter-hedonic model. Interestingly, in a recent study depressed patients with anhedonia outperformed depressed patients without it on a reward learning task (Min et al. 2024). This is consistent with the present model (Fig. 3), which suggests that in the absence (or significantly reduced) raw desire, as is the case in anhedonia, the agent's GM relies more on contextualized desires and thus may learn particular rewards more accurately.

The contrasting perspectives on OCD and depression as, respectively, hypo- and hyperfunctional deferred action explain the contrasting behavioral interventions for these conditions. In OCD, it is response prevention (Reid et al., 2021), whereas it is behavioral activation for depression (Dimidjian et al., 2006). The apparent wisdom of these approaches is that by simply engaging in or deferring actions, the patients increase their confidence (precision) in those policies, which can then generalize to their meta-confidence in action vs inaction and lead to adjustment of their GM’s parameters.

Framing (raw) desire as inference on affect (Fig. 3) leads to certain conclusions about psychopathology. Because of the desire-affect link, affective disorders can be considered motivational and vice versa. In the above examples, OCD and depression are both as evidenced by anxiety and mood disturbances in OCD (Goodwin, 2015) and loss of motivation in depression (DSM-5 TR). Another conclusion is that disordered motivational-affective states are subjectively desired, although not enjoyed. This is because desire, as discussed earlier, can make counter-hedonic inferences as long as they serve the EFE minimization. Thus, in depression, patients desire inaction and in OCD compulsion. This conclusion reflects the “optimal inference with suboptimal models” view of psychopathology (Schwartenbeck, FitzGerald, Mathys, Wurst et al., 2015b).

## Conclusion and future directions

To summarize the main arguments of this paper, we suggest that the free-energy and evolutionary principles endow allostatic and autopoietic systems, which biological organisms are, with a belief, which is also motivation, in their existence and propagation. In organisms having a brain and affective capacity, this belief is felt, and motivation thus develops into desire. Owing to its domain-general property (at the most abstract level), desire equips organisms with a better ability to optimize their behavior in response to the multitude of affordances and threats to their existence, thus making it more robust. Mechanistically, desire is made possible as well as felt and conscious by deferred action. Accordingly, disorders of affect and motivation can be considered as dysregulation of desire and deferred action. This view overcomes the belief-desire dichotomy.

Intuitively, motivation is commonly understood as a will to act, leaving inaction with a passive role. Here, we treat inaction, or more specifically, deferred action as an active cognitive state and argue that to better understand desire and motivation, we need to consider deferred action’s mechanisms and function. To that end, we approach desire and deferred action from the active inference standpoint, drawing an analogy with the AIF mechanics of motor movement (Brown et al., 2013). The proposed model adds a nuance to the gaining in acceptance view of psychopathology as false inference (Friston, 2023) by interpreting pathology, such as OCD and depression, as disturbance of deferred action and desire. Such a view may provide additional insights for clinical practice.

Despite its implicit inclusion in the AIF formalism, deferred action has not, to our knowledge, been explicitly modelled in computer simulations. Such modelling could be useful for the exploration of the dynamics of motivation and decision-making. Better models of motivation could then find application in optimization of human activity. Should a sentient artificial agent be developed, equipping it with deferred action would enable it to self-regulate desire. Perhaps the most immediate utility of this line of study would be the development of a model of impaired deferred action for clinical research.

## Data Availability

Not applicable.

## Footnotes

1 The term motivation, as understood in AIF (and used in this paper), stands for the reason to act and applies to behavior at all levels of complexity from bacteria to man, which is different from its conventional psychological meaning usually reserved for a conscious disposition.

2 Strictly speaking, the second law only applies to closed systems. Its generalization to open systems—considered under the free-energy principle—is known as integral fluctuation theorems (Seifert, 2012).

3 The meaning of allostasis has been expanded to include its predictive quality, where an organism acts to adapt to an expected environmental challenge, which property likely evolved with the advent of multicellularity (Sterling, 2020).

4 Conation is used interchangeably with desire here to capture both its philosophical and psychological aspects.

5 From this perspective, motivation becomes conative, as it reaches the level of consciousness.

6 We would also suggest an intuitively plausible though not empirically supported state of mind, where one is aware of wanting something not yet knowing what that is; simply wanting to be in a state other than the current one, presumably with a more positive affect.

7 This question is related to the paradox of so called “dark room,” which is outside this paper’s focus but has been widely discussed in the predictive processing literature (Smith et al., 2022).

8 The view of psychopathology as optimal inference by a suboptimal model can be an answer to the ‘dark room’ paradox mentioned earlier. In a mental disorder, the individual is trapped in the cycle of minimizing the expected free energy of his generative model (either by action as in OCD, or inaction as in depression) by predicting and fulfilling outcomes that are contrary to his phenotype-typical needs.
